# Proteomics Analysis Reveals Novel RASSF2 Interaction Partners

**DOI:** 10.3390/cancers8030037

**Published:** 2016-03-16

**Authors:** Thibaut Barnoud, Daniel W. Wilkey, Michael L. Merchant, Jennifer A. Clark, Howard Donninger

**Affiliations:** 1 Department of Biochemistry and Molecular Genetics, University of Louisville, Louisville, KY 40202, USA; tfbarn02@exchange.louisville.edu; 2 Department of Medicine, James Graham Brown Cancer Center, Molecular Targets Program, University of Louisville, Louisville, KY 40202, USA; dwwilk02@exchange.louisville.edu (D.W.W.); michael.merchant@louisville.edu (M.L.M.); jennifer.clark@louisville.edu (J.A.C.)

**Keywords:** RASSF2, Vimentin, K-Ras, acetylation, SIRT1

## Abstract

RASSF2 is a tumor suppressor that shares homology with other Ras-association domain (RASSF) family members. It is a powerful pro-apoptotic K-Ras effector that is frequently inactivated in many human tumors. The exact mechanism by which RASSF2 functions is not clearly defined, but it likely acts as a scaffolding protein, modulating the activity of other pro-apoptotic effectors, thereby regulating and integrating tumor suppressor pathways. However, only a limited number of RASSF2 interacting partners have been identified to date. We used a proteomics based approach to identify additional RASSF2 interactions, and thereby gain a better insight into the mechanism of action of RASSF2. We identified several proteins, including C1QBP, Vimentin, Protein phosphatase 1G and Ribonuclease inhibitor that function in diverse biological processes, including protein post-translational modifications, epithelial-mesenchymal transition, cell migration and redox homeostasis, which have not previously been reported to interact with RASSF2. We independently validated two of these novel interactions, C1QBP and Vimentin and found that the interaction with C1QBP was enhanced by K-Ras whereas, interestingly, the Vimentin interaction was reduced by K-Ras. Additionally, RASSF2/K-Ras regulated the acetylation of Vimentin. Our data thus reveal novel mechanisms by which RASSF2 may exert its functions, several of which may be Ras-regulated.

## 1. Introduction

Activation of the Ras oncoprotein, in particular, K-Ras, is a key driving force in the development of multiple tumors [1,2,3]. Activated Ras stimulates multiple signaling pathways, including the Raf-MEK-ERK and PI3 kinase pathways, to promote transformation [4]. However, activated Ras can also inhibit cell proliferation and promote apoptosis and senescence [5,6,7]. While the growth-promoting pathways regulated by Ras are well characterized, the exact mechanisms by which activated Ras inhibits growth are not as well studied, but it is apparent that this involves interactions with death effectors, which facilitate the pro-death functions of Ras. The main Ras death effector proteins identified to date are the RASSF family of tumor suppressors [8,9].

The RASSF family of proteins consists of 10 family members (RASSF1-10) [8,9,10], all of which share a conserved RalGDS/AF6 Ras association (RA) domain either in the C-terminus (RASSF1-6) or N-terminus (RASSF7-10). However, only the classical family members (RASSF1-6) contain a conserved SARAH (Salvador/RASSF/Hpo) motif adjacent to the RA domain [8,9,10]. RASSF1A is the best-characterized family member and is arguably the most frequently inactivated tumor suppressor in human cancers [8]. It functions as a scaffolding protein that complexes with and modulates the activity of other pro-apoptotic effectors to regulate tumor suppressor pathways, such as the Hippo pathway [11]. Additionally, RASSF1A has functions in DNA damage repair [12,13], cell motility, cell cycle control and Bax-mediated apoptosis [14,15,16,17]. NORE1A (RASSF5), the other major RASSF family member, is also frequently inactivated in human cancers [8] and serves as a Ras effector connecting Ras to the Hippo pathway [18], is a key mediator of Ras-induced senescence [19,20], and links Ras to the control of protein stability [21].

RASSF2 is structurally related to RASSF1A and also serves as a K-Ras-specific effector in that it is found in an endogenous complex with K-Ras [22,23] and binds K-Ras in a GTP-dependent manner [24]. In fact, RASSF2 may be the most important Ras death effector; there is a positive correlation between RASSF2 methylation and K-Ras/BRAF mutations in primary tumors [25,26,27] and inactivation of RASSF2 enhances the transforming potential of K-Ras [22,26]. Like RASSF1A, RASSF2 is frequently inactivated in multiple human tumors by promoter methylation [28,29,30,31,32,33,34]. RASSF2 behaves as a tumor suppressor in that its overexpression promotes apoptosis and cell cycle arrest *in vitro* and inhibits tumor growth *in vivo* [24,28], and loss of RASSF2 expression results in enhanced invasion, growth in soft agar, and transformation [22,26]. RASSF2 may have functions in addition to regulating apoptosis and proliferation as RASSF2 knockout mice develop normally for the first two weeks but then develop growth retardation and die approximately 4 weeks after birth [35]. The exact mechanisms by which RASSF2 effects its growth inhibitory functions are not clearly defined. RASSF2 localizes predominantly to the nucleus [28,36] and thus is likely to have independent functions from RASSF1A, which is localized primarily in the cytoplasm. Similar to the other RASSF family members, RASSF2 has no intrinsic enzymatic activity or DNA binding properties, and acts as a scaffolding protein, modulating the functions of other pro-apoptotic effectors including the MST kinases [37,38] and PAR-4 [29].

In an attempt to gain a better understanding of the mode of action of RASSF2, we performed a proteomics analysis to identify further binding partners of RASSF2. Since RASSF2 is a *bona fide* Ras effector, we performed this analysis in the presence and absence of activated K-Ras to identify proteins whose interaction with RASSF2 was regulated by Ras. We identified several novel RASSF2 interactions, some of which were regulated by Ras, either positively or negatively, and others that appeared to be Ras independent. On further investigation, we found that these interactions were not limited to the nucleus, but also occurred in the cytoplasm, suggesting that RASSF2 may have functions outside of the nucleus. These data suggest that RASSF2 may regulate diverse biological processes within the cell, and implicate Ras in the control of several of these systems.

## 2. Results

### 2.1. Identification of RASSF2 Interacting Partners

RASSF2 likely functions in a similar manner to the other more well characterized RASSF family members by acting as a scaffolding molecule. While there is some homology between RASSF2 and the other RASSF family members to suggest they may share common binding partners [9] such as the MST kinases [37,38], there is sufficient difference between the family members to suggest they have unique binding partners, and therefore, potentially independent functions. In an attempt to identify further RASSF2 binding partners, we performed a proteomic analysis of immunoprecipitations of RASSF2 transfected into HEK-293T cells. Since RASSF2 is a *bona fide* Ras effector that forms an endogenous complex with K-Ras [22,24], we performed the proteomic analysis in the presence and absence of activated K-Ras to identify interactions that were Ras-regulated. Specific proteins co-purifying with HA affinity tagged proteins were identified by mass spectrometry and compared between conditions using a label-free, normalized spectral counting method. In this approach, a relative abundance value termed “emPAI” was calculated by Scaffold and used for relative quantification. These values, typically ranging from 0 to 9, are based on the exponential form (em) of the Protein Abundance Index (PAI) with the PAI defined as the number of observed peptides divided by the number of all possible tryptic peptides from a particular protein observable within the observable mass range [39]. We identified a number of known RASSF2 interacting proteins, such as the MST kinases, but also a number of novel interacting proteins. A select list, based on their biological activities, which have not previously been ascribed to RASSF2, is shown in Table 1. Some of these interactions were regulated by Ras, either in a positive or a negative manner, and some were Ras independent.

While the presence of RASSF2 as the most abundant protein in the reaction as well as the identification of several known RASSF2 binding partners validates our proteomics analysis, we have begun to confirm these data by Western blot analysis, and our initial progress indicates that these results may be accurate.

### 2.2. RASSF2 Interacts with C1QBP

**Figure 1:**
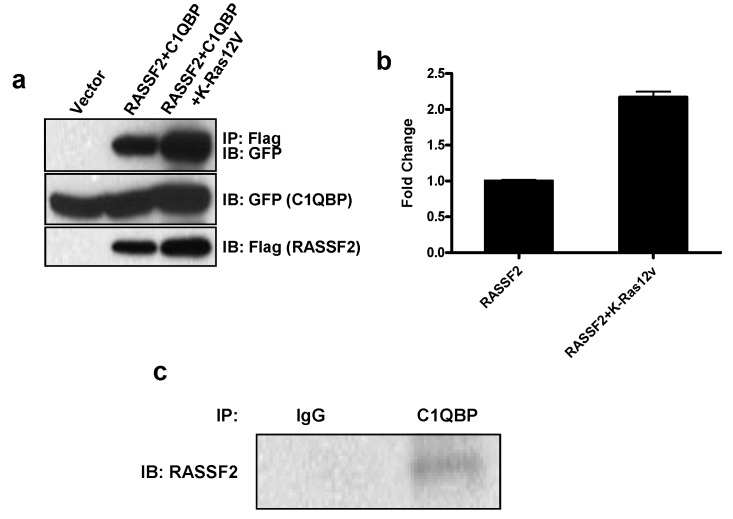
RASSF2 interacts with C1QBP. (**a**) HEK-293T cells were co-transfected with expression constructs for RASSF2, C1QBP and activated K-Ras for 24 hours. Cells were lysed and equal amounts of protein were immunoprecipitated with anti-Flag. The immunoprecipitates were analyzed by Western blotting using anti-Flag and anti-GFP antibodies. A representative blot is shown; (**b**) The density of the bands was quantitated using ImageJ software and amount of C1QBP found in complex with RASSF2 was determined after normalization to C1QBP expression; (**c**) Lysates from H441 lung cancer cells were immunoprecipitated with anti-C1QBP and immunoblotted with anti-RASSF2.

To confirm that RASSF2 interacts with C1QBP we performed co-immunoprecipitation experiments in HEK-293T cells co-transfected with RASSF2 and C1QBP in the presence and absence of activated Ras. We found that RASSF2 does complex with C1QBP, and furthermore, the interaction was enhanced in the presence of activated Ras (Figure 1a). On quantitation, we found Ras enhanced the C1QBP/RASSF2 interaction by approximately 2 fold (*p* < 0.05). Further analysis confirmed that endogenous C1QBP could be immunoprecipitated with endogenous RASSF2 from H441 lung cancer cells, which contain an activated Ras [24] (Figure 1b), suggesting the interaction is physiologically relevant.

**Figure 2:**
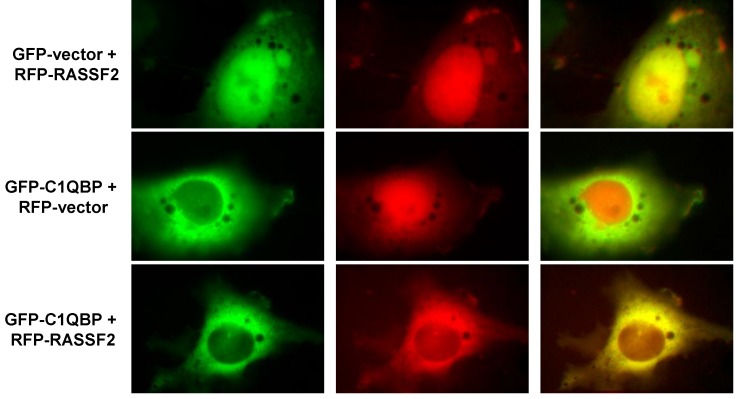
RASSF2 and C1QBP co-localize in mammalian cells. COS-7 cells were co-transfected with RFP-vector or RFP-RASSF2 and GFP-vector or GFP-C1QBP, and images were captured 24 hours later with a fluorescence microscope. Magnification (all images), 100×.

Having established that RASSF2 and C1QBP interact, we next sought to determine where in the cell this was occurring. RASSF2 shuttles between the cytoplasm and nucleus but is predominantly localized to the nucleus [24,28] and C1QBP is found in multiple compartments within the cell including the cytosol, cell membrane and nucleus, but is predominantly mitochondrial [40,41,42,43,44]. We transfected COS-7 cells with RFP-RASSF2 and GFP-C1QBP and analyzed the subcellular localization of the proteins by fluorescence microscopy. Consistent with other studies, we found that RASSF2 was predominantly localized to the nucleus [24,28,36] whereas C1QBP was localized predominantly in the cytoplasm (Figure 2). However, in cells transfected with both proteins, RASSF2 co-localized with C1QBP in the cytoplasm as evidenced by the yellow color in Figure 2 (bottom panel).

### 2.3. RASSF2 and Vimentin Interact

**Figure 3:**
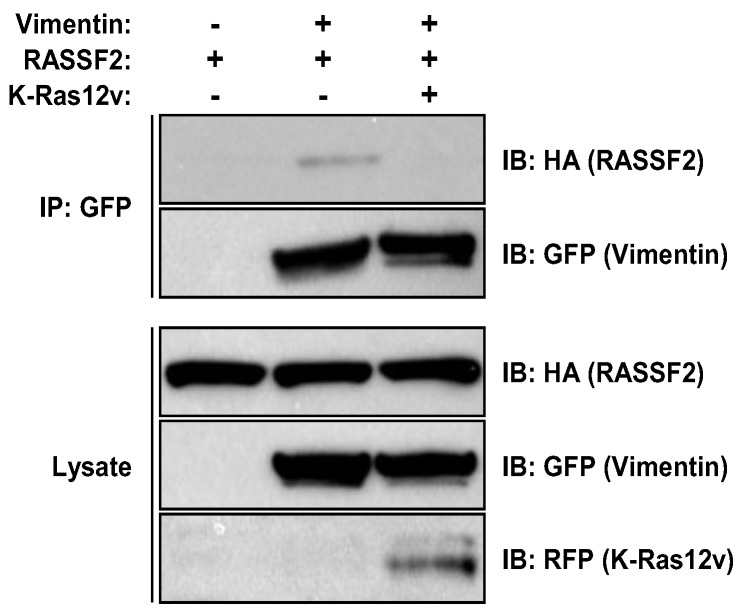
RASSF2 interacts with Vimentin. HEK-293T cells were co-transfected with HA-RASSF2 and GFP-Vimentin in the presence and absence of activated K-Ras. Equal amounts of lysates were immunoprecipitated with anti-GFP, fractionated on SDS gels and immunoblotted (IB) with anti-HA, anti-GFP and anti-RFP antibodies. Activated K-Ras reduces the interaction between RASSF2 and Vimentin.

In order to further confirm that our proteomics data was accurate, we chose to validate the interaction between RASSF2 and another potential binding partner identified in the screen, Vimentin (Table 1). Vimentin is a protein that is found in intermediate filaments (IFs) that stimulates cell migration and invasion [45], and is a key contributor to epithelial-mesenchymal-transition (EMT), a crucial early stage in the metastatic process [46,47,48]. To confirm that RASSF2 and Vimentin interact, we co-transfected HEK-293T cells with HA-tagged RASSF2 and GFP-tagged Vimentin in the presence and absence of activated K-Ras and performed co-immunoprecipitation experiments. The results showed that RASSF2 and Vimentin did indeed interact when exogenously expressed, and the interaction is abolished by activated K-Ras (Figure 3), confirming the results of the proteomics screen (Table 1).

**Figure 4:**
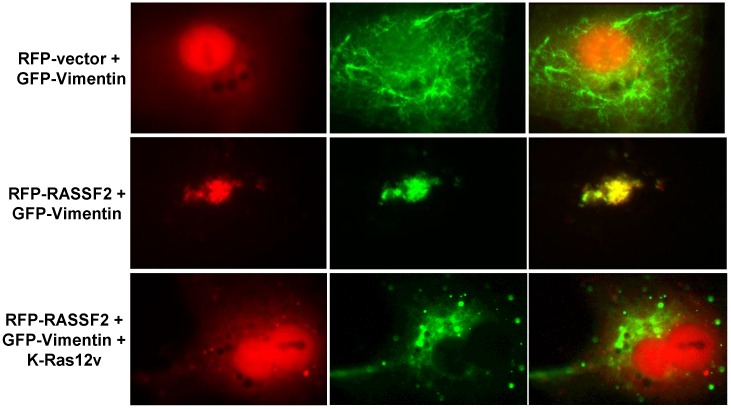
RASSF2 and Vimentin co-localize in the nucleus. COS-7 cells were co-transfected with RFP-RASSF2 and GFP-Vimentin in the presence and absence of activated K-Ras and fluorescent images captured 24 hours later. Magnification, 100×.

To determine where in the cells Vimentin and RASSF2 were interacting, we co-transfected COS-7 cells with RFP-RASSF2 and GFP-Vimentin and analyzed the cells by fluorescence microscopy. In the absence of RASSF2, Vimentin localized to fine filaments throughout the cytoplasm (Figure 4). In the presence of RASSF2, Vimentin co-localized with RASSF2 in the nucleus (Figure 4). However, in the presence of activated Ras, Vimentin no longer co-localized with RASSF2 (Figure 4), confirming both the proteomics and Western blot data.

### 2.4. RASSF2 and K-Ras Regulate Vimentin Acetylation

**Figure 5:**
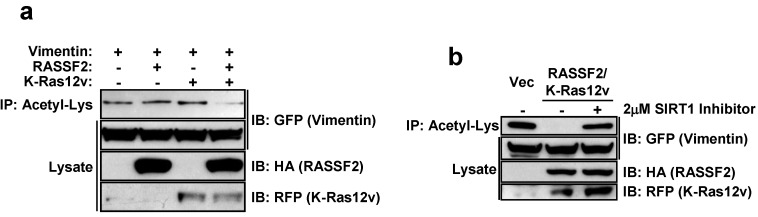
RASSF2 modulates Vimentin acetylation. (**a**) HEK-293T cells were transiently co-transfected with expression constructs for GFP-tagged Vimentin, HA-tagged RASSF2 and KATE-tagged K-Ras12v. Twenty-four hours later, the cells were lysed and lysates immunoprecipitated (IP) with anti-acetyl-Lys beads. The immunoprecipitates were resolved on SDS polyacrylamide gels and immunoblotted (IB) with anti-GFP, -HA and –RFP antibodies; (**b**) HEK-293T cells were transfected as described for panel (**a**). Thirty-six hours after transfection, 2 μM of the SIRT1 inhibitor, Inhauzin, was added to the cells. Sixteen hours later, cells were lysed and immunoprecipitated and immunoblotted as described for panel (**a**). Vec, cells transfected with vector control.

We have shown that the two major RASSF family members, RASSF1A and NORE1A (RASSF5) are able to regulate diverse biological functions within the cell by modulating protein post-translational modifications [12,19,20]. To determine if this is also the case for RASSF2, we analyzed the effects of RASSF2 on Vimentin acetylation. Vimentin is acetylated on several lysine residues [49], however the biological significance of acetylated Vimentin is not clear. Acetylated Vimentin appears to be clinically relevant though, in that acetylated Vimentin antibodies have been found in the sera of patients with rheumatoid arthritis [50]. We co-transfected HEK-293T cells with Vimentin and RASSF2 expression constructs in the presence and absence of activated Ras and measured effects on Vimentin acetylation by immunoprecipitating with an anti-Acetylated Lys antibody and immunoblotting for Vimentin. RASSF2 had no effect on Vimentin acetylation, however in the presence of activated K-Ras, RASSF2 dramatically reduced acetylated Vimentin (Figure 5a). We have recently shown that RASSF1A modulates XPA acetylation via the histone deacetylase SIRT1 [12] and SIRT1 regulates cell migration, invasion and the expression of EMT markers, including Vimentin [51]. Thus, we wondered whether RASSF2/K-Ras mediated Vimentin deactylation was also mediated by SIRT1. To determine the role of SIRT1 in the action of RASSF2/K-Ras, we used a specific SIRT1 inhibitor and showed that the ability of RASSF2/K-Ras to promote Vimentin deactylation is abolished when SIRT1 is inhibited (Figure 5b).

## 3. Discussion

While it is well established that RASSF2 is a *bona fide* K-Ras effector and powerful tumor suppressor, its modes of action are not well defined. It acts as a scaffolding protein in a similar manner to other members of the RASSF tumor suppressor family however the list of proteins it interacts with is not substantial. Its interaction with MST1 modulates MST1 kinase activity and results in activation of the JNK pathway and inactivation of the MST1-FOXO signaling pathway [38]. RASSF2 also interacts with MST2 resulting in stabilization of MST2 [37]. The biological significance of this effect has yet to be determined though. RASSF2 also interacts with the PAR-4 tumor suppressor and modulates its nuclear translocation, an essential process required for PAR-4 activity [29]. In addition to inducing apoptosis and inhibiting cell proliferation, RASSF2 may also function in processes regulating development as *RASSF2* knockout mice had defects in osteoblast differentiation resulting in defects in haematopoiesis and ultimately death [35]. Thus, we are beginning to gain some mechanistic insight into how RASSF2 functions, however, it is unlikely that the proteins that RASSF2 interacts with, and the pathways and processes it modulates, is limited to the ones described above.

In an effort to identify further proteins that interact with RASSF2 and uncover novel pathways and biological processes it may regulate, we performed a proteomics analysis of RASSF2 transfected into HEK-293T cells. Since RASSF2 is an established Ras effector, we performed the screen in the presence and absence of activated K-Ras to identify interactions that were regulated by Ras. In addition to known RASSF2 interacting partners, we identified several proteins that have not previously been shown to interact with RASSF2 (Table 1). Some of these interactions were regulated by Ras, either in a positive manner or negatively, and some were Ras-independent. The proteins identified in the proteomics screen are involved in a broad range of cellular processes, from apoptosis to protein transport and epithelial-mesenchymal-transition (EMT). We have begun to validate these data by independent means and our initial observations suggest that the proteomics data is accurate.

We chose to confirm two of these novel interactions, C1QBP and Vimentin, by Western blot analysis, and found that for both proteins, the Western blot analysis supported the proteomics results. C1QBP is a multicompartmental protein that is predominantly localized to mitochondria but can also translocate to the nucleus [40,41,42,52]. Given that RASSF2 is predominantly nuclear and C1QBP is cytoplasmic/mitochondrial we used fluorescence microscopy to determine the cellular localization of the interaction, and found that it occurs in the cytoplasm. RASSF2 has previously been reported to interact with the MST kinases in the cytoplasm [37] and cytoplasmic RASSF2 retains its tumor suppressor activity [53]. The function of C1QBP is still unclear but it appears to play a role in tumorigenesis, although whether this role is tumorigenic or tumor suppressive is not clearly defined. The expression of C1QBP is up-regulated in human tumors and correlates with poor prognosis in breast and prostate cancer [43,54,55] and over-expression of C1QBP results in enhanced cell proliferation [56] consistent with it being pro-tumorigenic. Conversely, C1QBP has been reported to be required for mitochondrial-dependent apoptosis mediated by p14^ARF^ and cisplatin [57,58]. One possible function of the interaction between RASSF2 and C1QBP is the regulation of atypical PKC (aPKC) activity. RASSF2 forms a complex with both PAR-4 [29] and C1QBP (figure 1), both of which can interact with the aPKCs [59,60]. While the interaction between PAR-4 and the aPKCs inactivates them [61], binding of C1QBP activates them [60]. Thus, there is the potential for RASSF2 to co-ordinately regulate the activity of the aPKCs by interacting with PAR-4 and C1QBP, and since RASSF2 binds directly to K-Ras, it may serve to couple K-Ras to this system. It will be interesting to determine how the interaction between RASSF2 and C1QBP affects the function of either protein and is the subject of ongoing research.

The other interaction we independently validated was that between RASSF2 and Vimentin. Vimentin is a type III intermediate filament protein whose expression is used as a canonical marker of EMT [46,47], but that also has functions in cell adhesion, migration and signaling [62]. Vimentin has been implicated in regulating signaling processes mediated by 14-3-3 proteins [62] and may act to inhibit the interaction of 14-3-3 with other target proteins, such as Raf [63]. Vimentin has been shown to interact with 14-3-3ε [64], and intriguingly, one of the novel RASSF2 binding partners identified in our proteomics screen was 14-3-3ε (Table 1). It is, thus, tempting to speculate the existence of a RASSF2/14-3-3/Vimentin complex, where RASSF2 scaffolds Vimentin to 14-3-3. Vimentin is acetylated on several lysine residues [49] and since we have previously found that RASSF1A and NORE1A can modulate protein post-translational modifications [12,19,20], we determined whether RASSF2 could also be regulating the acetylation of Vimentin, and indeed found that RASSF2/K-Ras induced de-acetylation of Vimentin, and this was SIRT1-dependent (Figure 5). Protein acetylation has been implicated in many biological processes by regulating protein interactions, protein activity and subcellular localization [65], and our data showing that RASSF2 can regulate Vimentin acetylation reveals a further mechanism by which RASSF2 can mediate its functions, and future work will be required to identify additional target proteins whose post-translational modifications are modulated by RASSF2.

In summary, to gain a better insight into the mechanism of action of the RASSF2 tumor suppressor, we have identified novel interactions between RASSF2 and proteins regulating diverse biological activities. In addition to its potential role in EMT and regulating 14-3-3 signaling processes through its interaction with Vimentin, and regulating C1QBP function, our proteomics data suggest that RASSF2 may have roles in regulating the activity of chaperones (DnaJ homolog subfamily A member 1), in mediating intracellular redox homeostasis (through its interaction with ribonuclease inhibitor 1 [66]) and in modulating protein phosphorylation through interacting with protein phosphatase 1G. Since RASSF2 is a powerful Ras effector, and several of these interactions are regulated by Ras, our data reveal potential novel functions for Ras not previously ascribed to Ras biology.

## 4. Materials and Methods

### 4.1. Cell Lines and Culture Conditions

HEK-293T and COS-7 cells were maintained in DMEM (Corning Cellgro, Manassas, VA, USA) supplemented with 10% fetal bovine serum (FBS; Valley Biomedical Inc., Winchester, VA, UAS) and 1% penicillin-streptomycin (Corning Cellgro).

### 4.2. Plasmids and Transfections

HA-tagged RASSF2 was generated by cloning a BamHI/MfeI RASSF2 cDNA fragment into a pcDNA vector with an HA epitope tag as previously described [24]. RFP-tagged RASSF2 and KATE-tagged K-Ras12V expression constructs have been previously described [19,29] and green fluorescent protein (GFP) tagged Vimentin was a gift from Michael Davidson (Addgene, Cambridge, MA, USA) (plasmid # 56439), GFP-C1QBP was constructed by PCR amplifying the C1QBP cDNA (obtained from GeneCopoeia, Rockville, MD, USA) with primers containing a 5ʹ BamHI and a 3ʹ EcoRI restriction site and cloning the PCR product into pEGFP-C1 (Clontech, Mountain View, CA, USA) digested with BglII and EcoRI. Exponentially growing cells were transfected with 2 μg DNA using jetPRIME^®^ transfection reagent (Polyplus, Illkirch, FR) as per the manufacturer’s instructions.

### 4.3. Western Blots and Immunoprecipitation

Total cell lysates were prepared by lysing the cells in RIPA buffer (1% NP40; 50 mM Tris, pH 7.5; 150 mM NaCl) supplemented with a protease inhibitor cocktail (Sigma, St. Louis, MO, USA) and 1 mM sodium orthovanadate. Immunoprecipitations were performed using GFP conjugated sepharose beads (Allele Biotechnology, San Diego, CA, USA), mouse monoclonal HA beads (Sigma-Aldrich) and acetyl-lysine conjugated agarose beads (Immunechem, BC, Canada). Anti-GFP antibody was obtained from Santa Cruz Biotechnology (Santa Cruz, CA, USA), anti-HA antibody from Covance and anti-RFP antibody from Evrogen (Moscow, Russia). HRP conjugated Trueblot secondary antibodies were purchased from eBioscience (eBioscience Inc., San Diego, CA, USA) and Western blots were developed using West Pico or West Femto enhanced ECL detection system (Thermo Scientific, Rockford, IL, USA).

### 4.4. Proteomic Sample Handling

Immunoprecipitation complexes and beads were washed using cold PBS, eluted using 40 µL of 4% SDS/0.1 M Tris-HCl Ph 8.5 and 5.55 µL of 1 M DTT with heating at 95 °C for 5 min. After cooling to room temperature, 3 volumes of 8 M urea/0.1 M Tris-HCl pH 8.5 was added to each sample, and the eluate was filtered through a 0.45 µm cellulose acetate filter to ensure removal of beads and bead debris. The filtrate was transferred to a Microcon-10 Ultracel YM-10 (10,000 NMWL centrifugal filter, Millipore, Bedford, MA, USA) and digested in solution using 0.1 µg of trypsin (Promega, Madison, WI, USA) according to the filter assisted sample preparation method of Wisniewski *et al.* [67]. The digested, ultra-filtered samples were trap-cleaned with C18 PROTO^TM^, 300 Å Ultra MicroSpin columns, lyophilized by vacuum centrifugation, and redissolved into 16 µL of 2% v/v acetonitrile for analysis by 1 dimension (1D) liquid chromatographic mass spectrometric (LCMS) analysis.

### 4.5. LCMS Data Acquisition

Data were collected using a Waters nanoAcquity M-Class UPLC^®^ (Waters, Milford, MA, USA) with a 180 µm × 20 mm (Symmetry^®^ C18, 5 µm, 100 Å) trap and a Waters Acquity UPLC^®^ M-Class 75 µm × 150 mm (HSS T3 C18, 1.8 µm) separating column prior to nanoelectrospray into a Synapt G2-Si mass spectrometer (Waters, Milford, MA, USA) was used to collect data from the LC eluate. A Fast DDA acquisition method was created in MassLynx v4.1 SCN924 (Waters, Milford, MA, USA). The spectrometer was operated in positive polarity in resolution mode. A 0.2 s continuum MS survey scan was taken from 100 to 2000 Da. For up to five ions with a threshold of 500, the instrument was switched to MS/MS, and a 0.2 s continuum scan was taken from 50 to 2000 Da. Ions were fragmented in the trap region using a collision energy ramp from 15 to 40 V. Peak detection was used to limit MS/MS acquisition to only 2+, 3+, and 4+ de-isotoped peaks. Dynamic peak exclusion was used to exclude peaks within ±2500 mDa for 30 s following MS/MS acquisition and leucine enkephalin (556.2771 Da/e) was used as the lock mass during data acquisition.

### 4.6. Analysis of LCMS Datasets

The RAW files were processed as an Electrospray DDA experiment in ProteinLynx Global Server^TM^ 3.0.2 (Waters, Milford, MA, USA). Both the electrospray survey and MSMS scans were calibrated to the 556.2771 Da/e leucine enkephalin lock mass (two lock spray scans with a lock mass tolerance of 0.1 Da). Adaptive noise reduction was used, and the deisotoped, processed spectra were exported as an mgf file for loading into Proteome Discoverer v1.4.1.114 (Thermo, Rockford, IL, USA) for analysis. The database used in Mascot v2.5.1 and SequestHT searches was the 10/29/2015 version of the UniprotKB *Homo sapiens* reference proteome canonical and isoform sequences. The database was annotated with the FASTA sequences pmKATE2-n vector, RASSF2 with HA tag, K-Ras with pmKATE2-n tag, and the nonhuman sequences from the 1/1/2012 version of the gpm.org cRAP database were appended to it. Mass tolerances of 100 ppm for precursors and 0.1 Da for fragments were used in both search algorithms. In order to initiate a reverse database search, a Target Decoy PSM Validator node was included in the workflow. The resulting msf files from Proteome Discoverer were loaded into Scaffold Q+S v4.4.5 (Proteome Software, Portland, OR, USA). The false discovery rate for peptides was calculated using the Scaffold Local FDR algorithm. Protein probabilities were calculated using the Protein Prophet algorithm. Proteins were grouped by Scaffold protein cluster analysis to satisfy the parsimony principle.

## 5. Conclusions

Our data identify novel binding partners for RASSF2 and reveal potential novel mechanisms by which RASSF2 exerts its functions.

## Figures and Tables

**Table 1:** Select list of proteins interacting with RASSF2.

Protein	−K-Ras12v	+K-Ras12v
RASSF2	9.08 ^a^	9.09
MST1	0.72	1.03
MST2	0.46	0.23
C1QBP	0.61	1.35
Vimentin	0.35	0
14-3-3ε	0.59	0.88
DnaJ homolog subfamily A member 1	0.24	0.61
Ribonuclease inhibitor	0.42	0.63
Ankyrin repeat and KH domain-containing protein 1	0.1	0.15
Protein phosphatase 1G	0.09	0.17

^a^ Quantitative value (normalized emPAI). Proteins in blue are proteins previously been reported to interact with RASSF2.

## Abbreviations

The following abbreviations are used in this manuscript:

RASSF2: Ras association (RalGDS/AF-6) domain family member 2
EMT: Epithelial-to-mesenchymal transition
Raf: Raf-1 proto-oncogene, serine/threonine kinase
MEK: Mitogen-activated protein kinase
ERK: Extracellular regulated MAP kinase
SIRT1: Sirtuin 1
GFP: Green fluorescent protein
RFP: Red fluorescent protein
